# A meta-analysis of long follow-up outcomes of laparoscopic Nissen (total) *versus* Toupet (270°) fundoplication for gastro-esophageal reflux disease based on randomized controlled trials in adults

**DOI:** 10.1186/s12876-016-0502-8

**Published:** 2016-08-02

**Authors:** Xing Du, Zhiwei Hu, Chao Yan, Chao Zhang, Zhonggao Wang, Jimin Wu

**Affiliations:** 1 Department of Vascular Surgery, Xuan Wu Hospital, Capital Medical University, Beijing, 100053 China; 2 Department of Gastroesophageal Reflux Disease, the General Hospital of the PLA Rocket Force, Beijing, 100088 China

**Keywords:** Laparoscopic fundoplication, Nissen, Toupet, Gastro-esophageal reflux disease, Randomized controlled trials, Meta-analysis

## Abstract

**Background:**

Laparoscopic Nissen fundoplication (LNF) is the most common surgical procedure for the surgical management of gastro-esophageal reflux disease (GERD). Laparoscopic Toupet fundoplication (LTF) has been reported to have a lower prevalence of postoperative complications yet still obtain a similar level of reflux control. We conducted a meta-analysis to confirm the value of LNF and LTF.

**Methods:**

PubMed, Medline, Embase, Cochrane Library and Springerlink were searched for randomized controlled trials (RCTs) comparing LNF and LTF. Data regarding the benefits and adverse results of two techniques were extracted and compared using a meta-analysis.

**Results:**

Eight eligible RCTs comparing LNF (*n* = 625) and LTF (*n* = 567) were identified. There were no significant differences between LNF and LTF with regard to hospitalization duration, perioperative complications, patient satisfaction, postoperative heartburn, regurgitation, postoperative DeMeester scores, or esophagites. A shorter operative time and higher postoperative lower esophageal sphincter pressure were associated with LNF. Prevalence of postoperative dysphagia, gas-bloating, inability to belch, dilatation for dysphagia and reoperation were higher after LNF, but subgroup analyses showed that differences with respect to dysphagia between LNF and LTF disappeared over time. Subgroup analyses did not support “tailored therapy” according to preoperative esophageal motility.

**Conclusions:**

LNF and LTF have equivalently good control of GERD and result in a similar prevalence of patient satisfaction. Based on current evidence, it is not rational or advisable to abandon LNF when choosing a surgical procedure for GERD.

## Background

Gastro-esophageal reflux disease (GERD) is one of the most common gastrointestinal diseases in which the gastric contents flow into the esophagus through the incompetent lower esophageal sphincter (LES) and can cause troublesome symptoms and complications [1]. According to epidemiology studies, GERD affects 10–28 % of Europeans [2] and 3–7 % of Asians [3] (with an increase in prevalence in some Asian countries), which leads to a considerable healthcare burden and low quality of life.

Currently, the therapy approaches of GERD include lifestyle modifications, proton pump inhibitor-based pharmacologic therapy and surgical intervention [4]. Fundoplication has achieved an established role in the management of complicated GERD [5–7]. In the last two decades, many studies including randomized controlled trials (RCTs) and meta-analyses have shown that laparoscopic surgery is as effective and safe as open surgery for the treatment of GERD, while reducing the hospital stay and incidence of complications [8–10].

There are two major anti-reflux procedures: 360° total (Nissen) fundoplication and 270° partial (Toupet) fundoplication. Currently laparoscopic Nissen fundoplication (LNF) is the most common surgical procedure for the management of GERD offering promising long-term outcomes [11] and has been recommended as a choice of surgical therapy by the European Study Group for Antireflux Surgery and the Society of American Gastrointestinal Endoscopic Surgeons [12]. Nevertheless, LNF can induce functional disorders, such as dysphagia, gas-bloating and an inability to belch. Compared to LNF, several surgeons have stated that laparoscopic Toupet fundoplication (LTF) has a lower prevalence of postoperative complications while obtaining a similar control of reflux [13], but, several studies have failed to show a significant difference between them [14, 15]. Also, whether preoperative esophageal motility (EM) should be considered when surgeons select a procedure has not been elucidated. Hence, the controversy regarding the optimal surgical method continues.

Several systematic reviews and meta-analyses have compared outcomes between laparoscopic partial fundoplication and LNF up until 2013. However, generalization of all types of partial fundoplication into one category in a review is not appropriate [16–18]. In the past 3 years, several RCTs [19–21] published comparison between the value of LNF and LTF. Hence, it is necessary to synthesize data from those RCTs with existing RCTs in a meta-analysis to re-evaluate outcomes so that evidence for optimal clinical practice can be provided.

## Methods

This meta-analysis was conducted and the results were described according to the PRISMA statement [22].

### Search strategy

Following electronic databases were searched till October 2015: PubMed, Medline, Embase, Cochrane Library (issue 10, 2015) and Springerlink. A manual search was also performed to identify trials in the reference lists of the articles acquired. Only articles written in English were searched. A search strategy using disease-specific terms (e.g., gastro-esophageal reflux disease), management-specific terms (e.g., laparoscopic anti-reflux fundoplication) and terms related to surgical procedures (e.g., Nissen, Toupet, total and partial) were adopted.

### Inclusion criteria and exclusion criteria

Inclusion criteria were: (i) RCTs comparing efficacy and adverse outcomes of LNF and 270° LTF; (ii) age ≥16 years; (iii) laparoscopic procedure was carried out in all patients; (iv) duration of follow-up ≥12 months; (v) raw data could be extracted from studies to calculate outcomes; (vi) patients were diagnosed definitively preoperatively.

Exclusion criteria were: (i) non-RCTs; (ii) trials comparing total and non-posterior partial fundoplication (e.g., total *vs.* anterior fundoplication); (iii) fundoplications were carried out with laparotomy; (iv) trials involving patients aged <16 years; (v) studies published repeatedly in different journals; (vi) studies for which raw data could not be extracted to obtain pooled results and the corresponding author could not provide data requested.

### Outcomes of interest and definitions

Outcome parameters were described as below. Subjective evaluation: patient satisfaction with the intervention, postoperative heartburn and regurgitation (defined as subjective persistence of reflux and/or recurrence on a dichotomous scale compared with the preoperative state). Objective evaluation: DeMeester scores on 24-h pH monitoring, LES pressure, and endoscopic esophagitis. Prevalence of perioperative complications, postoperative complications, postoperative dilatation for dysphagia, reoperation, operating time, duration of hospitalization, and mortality were also evaluated. Among the outcomes mentioned above, patient satisfaction, postoperative heartburn and dysphagia were regarded as primary outcome parameters, and the others were regarded as secondary outcome parameters.

### Data extraction

Two reviewers extracted details from selected studies independently. Data comprised (i) information provided and the quality of the research: first author, publication year, study population characteristics, study design, sample size, follow-up duration, and inclusion/exclusion criteria; and (ii) outcomes analysis, including beneficial and adverse results. Disagreements between reviewers were resolved by discussion and consensus. If data were missing, the authors of the original studies were contacted to provide the relevant information. Outcomes of interest of repeated RCTs in which the study population arose from the same cohort published in different journals at different phases were extracted based on the article that was published most recently.

### Statistical analysis

Data extracted from eligible trials were integrated with Review Manager 5.3 provided by the Cochrane Collaboration. Outcomes reported by two or more studies were pooled in the meta-analysis. Dichotomous and continuous outcomes were presented as risk ratio (RR) and standard mean difference (SMD) respectively. Dichotomous outcomes were pooled using the Mantel-Haenszel method, while continuous outcomes were pooled using the inverse variance method. The fixed-effects model was used if heterogeneity was absent (χ^2^ test, *P* > 0.1 and *I*^*2*^ < 50 %); otherwise the random-effects model was used [23]. If excessive heterogeneity was present, data were first rechecked. If heterogeneity persisted, sensitivity or subgroup analyses were undertaken to explore its causes. Subgroup analysis was performed to assess the impact of follow-up duration and EM.

### Quality assessment

According to Cochrane criteria guidelines, all included studies were evaluated to ascertain if methodological bias was present [24].

## Results

### Description of studies

**Fig. 1:**
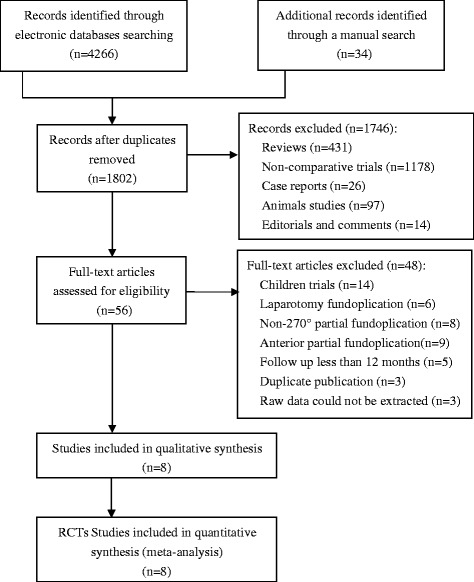
A flow chart showing the process and result of trials screening. RCTs, randomized controlled trials

After screening of trials according to inclusion and exclusion criteria, eight RCTs [19–21, 25–29] were identified, including 1192 patients, of whom 625 (52.43 %) underwent LNF and 567 (47.57 %) underwent LTF (Fig. 1). Included studies were published between 2003 and 2015. Duration of follow-up ranged from 12 to 60 months. Not all studies provided data regarding outcomes of interest. In two trials [19, 27], whether the baseline information of two groups was similar was not clear. Basic characteristics of included RCTs are listed in Table 1.

**Table 1:** The basic characteristics of included randomized clinical trials

Source	Country	N	Sex ratio	Age (years)	BMI or Weight (Kg)	DSGV	Follow–up
		(LNF/LTF)	(Male/Female)	(LNF/LTF)	(LNF/LTF)		(months)
Chrysos 2003[25]	Greece	14/19	18/15	61.7 ± 8.7/59.2 ± 11.5	NR	NO	12
Guérin 2007[26]	Belgium	77/63	86/54	NR	Mean BMI 27.9/27.2	YES	36
Strate 2008[27]	Germany	100/100	121/79	56(20–80)	Median BMI 26.4(18.9–40.4)	YES	24
Booth 2008[28]	England	64/63	84/43	45.3(21–86)/44.2(19–69)	Mean Weight 81.6/80.2	YES	12
Shaw 2010[29]	South Africa	50/50	60/40	45.2(28–72)/45.6(25–67)	Mean BMI 29.3 ± 5.2/29.2 ± 5.2	YES	60
Qin 2013[19]	China	215/168	194/189	56.3(34–82)	NR	NO	12
Koch 2013[20]	Austria	62/63	78/47	50.32(20–76)/51.87(25–81)	Mean BMI 28.18(19.47–41.80)/27.32(19.66–3.86)	YES	12
Wang 2015[21]	China	43/41	44/40	57 ± 13.2/57 ± 10.8	Mean BMI 23.5 ± 2.7/2.5 ± 3.4	YES	24

*LNF* laparoscopic Nissen fundoplication, *LTF* laparoscopic Toupet fundoplication, *BMI* body mass index, *DSGV* division of short gastric vessels, *NR* not report

### Quality assessment

The methodological quality of included trials is shown in Table 2. Main limitations resulted from poor description of randomization processes [19, 26], as well as a lack of (or poor description of) double-blinding processes [19, 20, 26].

**Table 2:** Risk of bias summary

	①	②	③	④	⑤	⑥	⑦
Chrysos 2003[25]	LR	LR	LR	LR	LR	LR	LR
Guérin 2007[26]	UR	UR	UR	LR	LR	LR	LR
Strate 2008[27]	LR	LR	LR	LR	LR	LR	LR
Booth 2008[28]	LR	LR	LR	LR	LR	LR	LR
Shaw 2010[29]	LR	LR	LR	LR	LR	LR	LR
Qin 2013[19]	HR	LR	UR	UR	LR	LR	LR
Koch 2013[20]	LR	LR	HR	LR	LR	LR	LR
Wang 2015[21]	LR	LR	LR	LR	LR	LR	LR

①:Random sequence generation; ②:Allocation concealment; ③:Blinding of participants and personnel ④:Blinding of outcomes assessment; ⑤:Incomplete outcome data; ⑥:Selective reporting; ⑦:Other bias. *LR* low risk, *UR* unclear risk, *HR* high risk

### In-hospital characteristics

#### Operating time

Six RCTs [19, 21, 25, 27–29] reported operating time for LNF and LTF. Because of significant heterogeneity, the random-effects model was used to pool data. Meta-analysis revealed a significant difference in this parameter between the two groups (SMD = –0.54 min, 95 % confidence interval (CI), –0.84 to –0.24 min, *P* = 0.0004) (Table 3). Operating time was shorter in patients undergoing LNF. Sensitivity analyses were done upon consideration of whether division of the short gastric vessels was a possible cause of heterogeneity. After removal of two studies [19, 25], heterogeneity disappeared and the result did not change (four studies with division of short gastric vessels: SMD = –0.34 min, 95 % CI, –0.51 to –0.16 min, *P* = 0.0001, χ^2^ test, *P* = 0.51, *I*^*2*^ = 0 %).

**Table 3:** Meta-analysis of some outcome parameters after LNF and LTF

Outcome	n		Heterogeneity test		Analysis model	SMD (95 % CI) or RR (95 % CI)	*P* value
	LNF	LTF	*I* ^*2*^	*P* value			
Operating time	486	441	77 %	0.0007	Random	SMD –0.54 (–0.84, –0.24)	0.0004
Duration of hospitalization	336	291	13 %	0.28	Fixed	SMD –0.05 (–0.41, 0.32)	0.8
Reoperation	274	282	4 %	0.37	Fixed	RR 3.16 (1.49, 6.68)	0.003
Preoperative DeMeester scores	484	441	0 %	0.45	Fixed	SMD 0.17 (0.02, 0.32)	0.02
Preoperative LES pressure	548	504	20 %	0.29	Fixed	SMD –0.06 (–0.21, 0.09)	0.43
Postoperative esophagitis	317	277	0 %	0.46	Fixed	RR 0.98 (0.81, 1.18)	0.8

*LNF* laparoscopic Nissen fundoplication, *LTF* laparoscopic Toupet fundoplication, *SMD* standard mean difference, *RR* risk ratio, *LES* lower esophageal sphincter

#### Duration of hospitalization

Four studies [19, 21, 25, 28] reported duration of hospitalization after LNF and LTF. Meta-analysis revealed no significant difference in this parameter between the two arms (SMD = –0.05 days, 95 % CI, –0.41 to 0.32 days, *P* = 0.80) (Table 3).

#### Perioperative complications

**Fig. 2:**
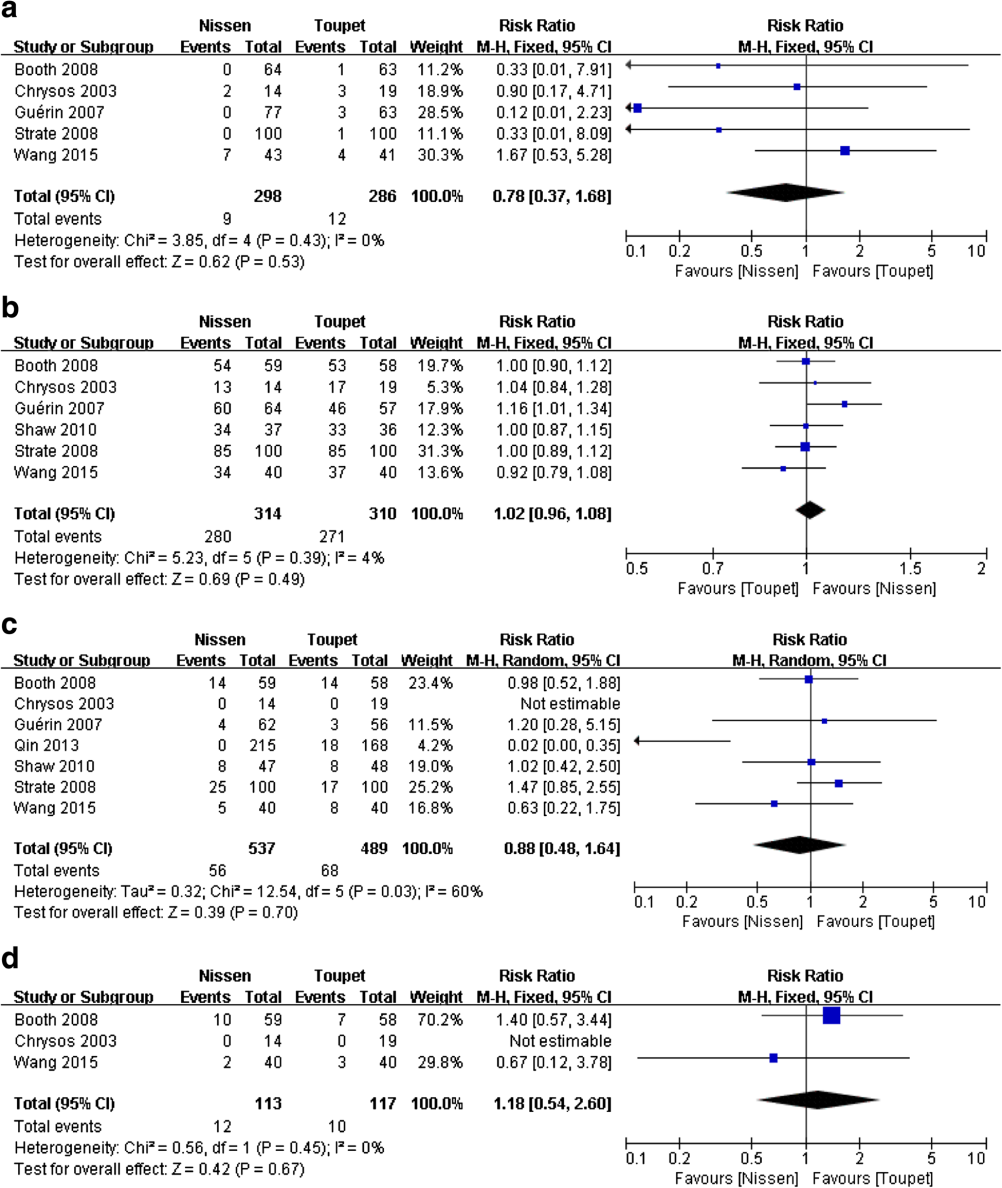
Meta-analysis of perioperative complications (**a**), patient satisfaction (**b**), postoperative heartburn (**c**), and regurgitation (**d**) after LNF and LTF

Five studies [21, 25–28] reported perioperative complications after LNF and LTF. Meta-analysis revealed no significant difference in this parameter between the two groups (RR = 0.78, 95 % CI, 0.37 to 1.68, *P* = 0.53) (Fig. 2).

### Patient satisfaction

Six trials [21, 25–29] reported patient satisfaction after LNF and LTF. Four RCTs counted the number of patients who were “very satisfied”/“satisfied” with the intervention. The other two RCTs employed a Visick scale to measure patient the degree of patient satisfaction. The Visick score ranged from I (excellent) to IV (poor) based on postoperative symptoms [30]. For this report, Visick scale grade I/II was defined as “very satisfied”/“satisfied” with the procedure. Meta-analysis revealed no significant difference in this outcome between the two arms (RR = 1.02, 95 % CI, 0.96 to 1.08, *P* = 0.49) (Fig. 2).

### Recurrence of GERD symptoms and prevalence of reoperation

#### Postoperative heartburn

Seven studies [19, 21, 25–29] reported postoperative heartburn after LNF and LTF. Excessive heterogeneity existed, so the random-effects model was used to pool data. Meta-analysis revealed no significant difference in this parameter between the two arms (RR = 0.88, 95 % CI, 0.48 to 1.64, *P* = 0.70) (Fig. 2). After removal of one RCT [19], heterogeneity was reduced significantly, and the result did not alter (RR = 1.11, 95 % CI, 0.79 to 1.57, *P* = 0.54, χ^2^ test, *P* = 0.67, *I*^*2*^ = 0 %).

#### Postoperative regurgitation

Three studies [21, 25, 28] reported postoperative regurgitation after LNF and LTF. Meta-analysis revealed no significant difference in this outcome between the two groups (RR = 1.18, 95 % CI, 0.54 to 2.60, *P* = 0.67) (Fig. 2).

#### Reoperation

Five studies [20, 25, 27–29] reported the prevalence of reoperation after LNF and LTF. Meta-analysis revealed a significant difference in this parameter between the two groups (RR = 3.16, 95 % CI, 1.49 to 6.68, *P* = 0.003) (Table 3). Findings showed a lower prevalence of reoperation after LTF.

### Objective outcomes

#### DeMeester scores

##### Preoperative DeMeester scores

Six studies [19–21, 25, 27, 29] reported preoperative DeMeester scores on 24-h pH monitoring. Meta-analysis revealed a significant difference in this parameter between the two arms (SMD = 0.17, 95 % CI, 0.02 to 0.32, *P* = 0.02) (Table 3). Slightly higher mean DeMeester scores before LNF were documented.

##### Postoperative DeMeester scores

**Fig. 3:**
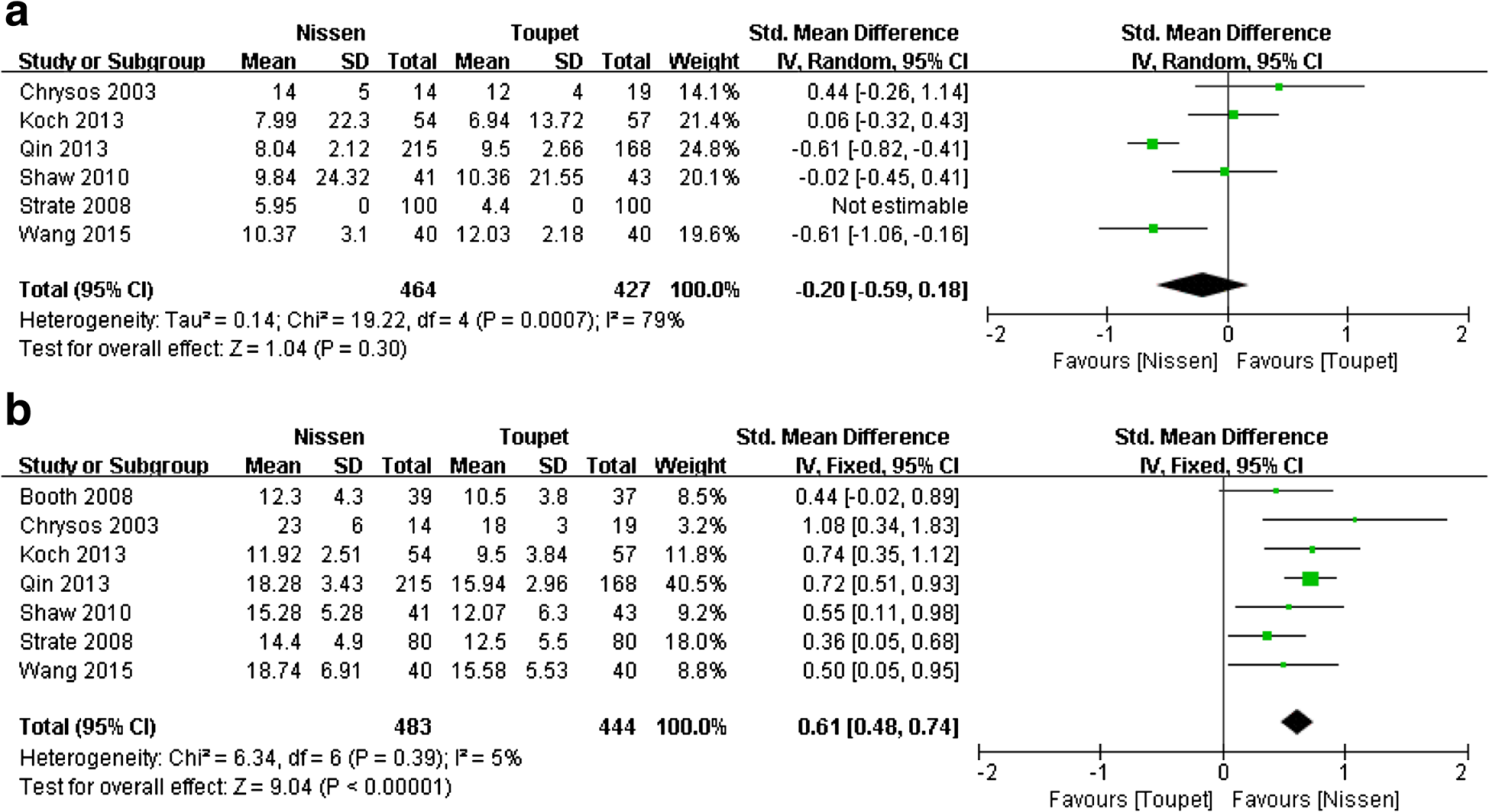
Meta-analysis of postoperative DeMeester scores (**a**) and LES pressure (**b**) after LNF and LTF

The same six studies [19–21, 25, 27, 29] mentioned above reported postoperative DeMeester scores on 24-h pH monitoring. Because of significant heterogeneity, the random-effects model was used to pool data. Meta-analysis revealed no significant difference in this parameter between the two arms (SMD = –0.20, 95 % CI, –0.59 to 0.18, *P* = 0.30) (Fig. 3).

#### LES pressure

##### Preoperative LES pressure

Seven studies [19–21, 25, 27–29] reported preoperative LES pressure. Meta-analysis revealed no significant difference in this parameter between the two groups (SMD = –0.06 mmHg, 95 % CI, –0.21 to 0.09 mmHg, *P* = 0.43) (Table 3).

##### Postoperative LES pressure

The same seven studies [19–21, 25, 27–29] mentioned above reported postoperative LES pressure. Meta-analysis revealed a significant difference in this parameter between the two groups (SMD = 0.61 mmHg, 95 % CI, 0.48 to 0.74 mmHg, *P* < 0.00001) (Fig. 3). The result suggested that LNF was associated with a higher mean postoperative LES pressure compared to LTF.

#### Postoperative esophagitis

Three studies [19, 25, 27] reported postoperative esophagitis after LNF and LTF. Meta-analysis revealed no statistically difference in this parameter between the two groups (RR = 0.98, 95 % CI, 0.81 to 1.18, *P* = 0.80) (Table 3).

### Postoperative complications

#### Postoperative dysphagia

Considering duration of follow-up and EM were possible factors influencing dysphagia, subgroup analyses were conducted based on these factors.

##### Subgroup analyses of dysphagia based on duration of follow-up

**Fig. 4:**
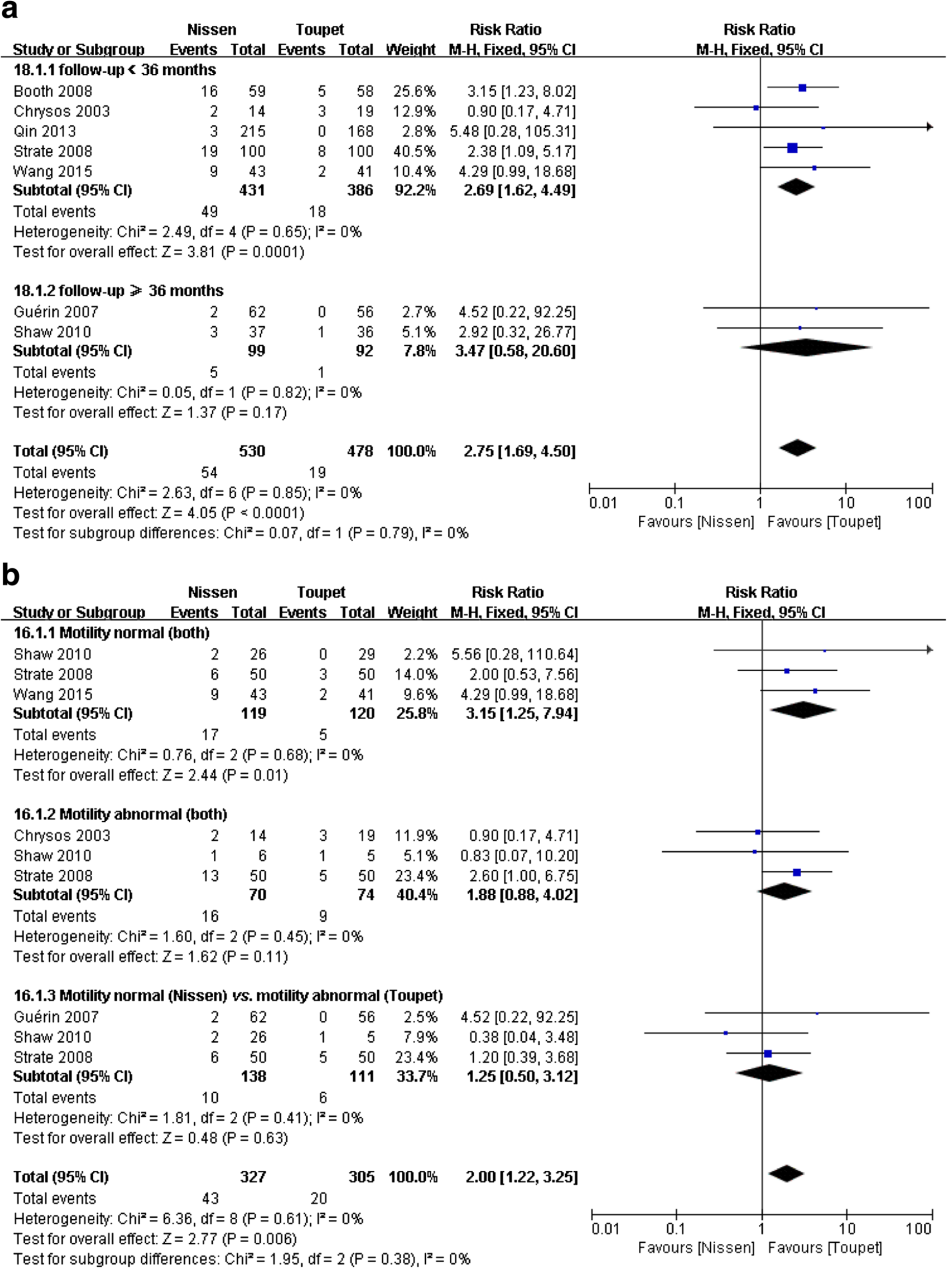
Subgroup analyses of postoperative dysphagia according to duration of follow-up (**a**) and preoperative EM (**b**)

Seven studies [19, 21, 25–29] reported postoperative dysphagia, including five studies [19, 21, 25, 27, 28] with duration of follow-up <36 months and two studies [27, 29] with duration of follow-up ≥36 months. When total-group analyses were performed, meta-analysis revealed a significant difference in this parameter between the two arms (RR = 2.75, 95 % CI, 1.69 to 4.50, *P* < 0.0001) (Fig. 4). Findings showed a lower prevalence of dysphagia for patients who had undergone LTF. In the subgroup with a follow-up <36 months, results also favored LTF (RR = 2.69, 95 % CI, 1.62 to 4.49, *P* = 0.0001) (Fig. 4). Nevertheless, in the subgroup with a follow-up ≥36 months, the prevalence of postoperative dysphagia was similar between the two groups (RR = 3.47, 95 % CI, 0.58 to 20.60, *P* = 0.17) (Fig. 4).

##### Subgroup analyses of dysphagia based on preoperative EM

In the subgroup with normal EM subgroup preoperatively, subgroup analyses revealed a significant difference in the outcomes between the two arms (subgroup 1: RR = 3.15, 95 % CI, 1.25 to 7.94, *P* = 0.01) (Fig. 4). The result showed a lower prevalence of dysphagia for patients who had undergone LTF. However, in the subgroup with abnormal EM or subgroup of LNF with normal EM and LTF with abnormal EM preoperatively, subgroup analyses failed to find a difference (subgroup 2: RR = 1.88, 95 % CI, 0.88 to 4.02, *P* = 0.11; subgroup 3: RR = 1.25, 95 % CI, 0.50 to 3.12, *P* = 0.63) (Fig. 4).

#### Postoperative dilatation for dysphagia

**Fig. 5:**
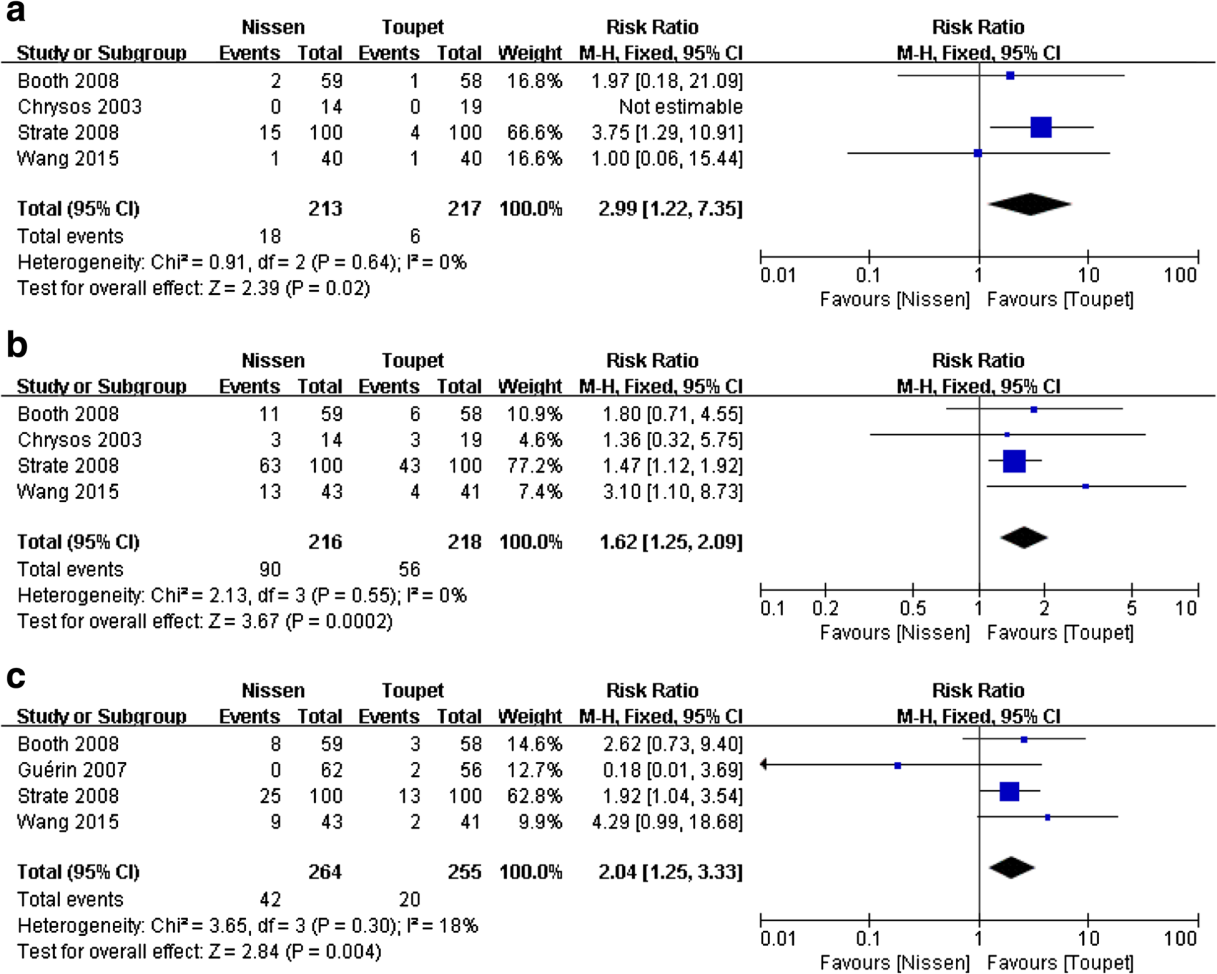
Meta-analysis of postoperative dilatation for dysphagia (**a**), gas-bloating (**b**) and inability to belch (**c**) after LNF and LTF

Four studies [21, 25, 27, 28] reported postoperative dilatation after LNF and LTF. Meta-analysis revealed a significant difference in this parameter between the two arms (RR = 2.99, 95 % CI, 1.22 to 7.35, *P* = 0.02) (Fig. 5). The result showed that LTF was associated with a slightly lower occurrence of dilatation for dysphagia compared to LNF.

#### Postoperative gas-bloating

Four studies [21, 25, 27, 28] reported postoperative gas-bloating after LNF and LTF. Meta-analysis revealed a significant difference in this parameter between the two arms (RR = 1.62, 95 % CI, 1.25 to 2.09, *P* = 0.0002) (Fig. 5). The result showed a lower occurrence of postoperative gas-bloating for patients who underwent LTF.

#### Postoperative inability to belch

Four studies [21, 26–28] reported postoperative inability to belch after LNF and LTF. Meta-analysis revealed a significant difference in this parameter between the two groups (RR = 2.04, 95 % CI, 1.25 to 3.33, *P* = 0.004) (Fig. 5). The result showed a lower occurrence of the inability to belch for patients who underwent LTF.

### Mortality

As no death was associated with these two surgical methods in hospital or during follow-up, the two arms could not be compared with regard to mortality.

### Sensitivity analysis

For two studies [19, 27], whether the baseline information of two arms was similar was not known. After removal of these two studies from the pooling analysis, the results did not alter substantially with regard to the primary outcomes: patient satisfaction, postoperative heartburn and postoperative dysphagia.

## Discussion

Since the first laparoscopic fundoplication was undertaken for GERD in 1991, laparoscopic surgery has been the “gold standard” for patients with medical-refractory GERD [31]. Although LNF is classic procedure for the treatment of GERD, it is challenged by LTF with less functional disorders.

In the last two decades, several RCTs have been conducted to compare LNF with LTF, but the results have been inconclusive. Between 2010 and 2015, four meta-analyses [32–35] comparing LNF with LTF were published. However, in the included trials, LTF was done with various circumferences (200°–270°), which would induce heterogeneity between trials and reduce the reliability and accuracy of the findings of the meta-analyses above. In addition, these meta-analyses neglected the comparability of baseline information of LNF and LTF. Several trials [19–21] of large-scale and long-term follow-up have been published in recent years, so re-evaluation and syntheses of data in existing trials are important.

According to the findings from the present meta-analysis, the following conclusions can be drawn. First, the prevalence of patient satisfaction was similar between LNF and LTF, and was high (LNF, 89.17 %; LTF, 87.42 %). Second, LTF was as effective as LNF with respect to symptom control. Third, the prevalence of postoperative dysphagia was higher after LNF, but with increasing duration of follow-up, the difference between two arms disappeared. Fourth, LNF was associated with higher LES pressure.

This report demonstrated that the operating time of LTF was longer than that of LNF, which might be due to the fact that the gastric fundus and both sides of the esophagus should be secured, respectively. Prevalence of perioperative complications between the two groups was not significantly different. But it is notable that Strate et al. [27] and Booth et al. [28] reported a perforation in the fundal wrap and an episode of perioperative bleeding after LTF. And Guérin et al. [26] also observed in-hospital bleeding. This phenomenon may be because the esophagus does not have a serosal layer and gastric fundus and both sides of the esophagus wall need to be sutured together, which may increase the risk of perforation and bleeding.

Heartburn and regurgitation are typical symptoms of GERD. Our report showed that LNF and LTF were similar with regard to reflux control. Importantly, objective parameters are not always in accordance with the symptoms or complaints of patients [27, 36, 37]. The value of laboratory examinations is limited for the diagnosis and evaluation of therapeutic efficacy for GERD, so the definition of “recurrent GERD” based on laboratory measurements alone may not be appropriate. Symptoms combined with objective parameters should be the main indications for surgical therapy, a view that is consistent with that of Tan et al [34].

With better control of GERD, LNF was previously regarded as the standard treatment for GERD. However, this concept has been challenged owing to postoperative functional disorders. We have shown that LNF is associated with a higher incidence of postoperative dysphagia, gas-bloating and inability to belch. However, subgroup analyses suggested that differences in the prevalence of dysphagia between two techniques disappeared over time. Furthermore, two decades follow-up results of a RCT [38] comparing open Nissen and Toupet demonstrated no difference in the prevalence of postoperative complications. As a reasonable and accurate index for assessing the efficacy of surgical treatment for GERD [39, 40], the patient satisfaction was high (≈90 %) and comparable between the two arms.

As for whether preoperative EM was an indication for “tailored therapy”, the subgroup analyses showed that EM was not correlated with postoperative dysphagia, indicating that “tailored therapy” according to EM was not indicated, which was consistent with other reports [30, 33]. It should be noted that the definition of EM in the included studies was not consistent, which might affect the ability to reach true conclusion.

With regard to reoperation, the most common causes were complications, recurrence of reflux symptoms, and others conditions. Specific data could not be obtained, so subgroup analyses based on causes could not be done.

For baseline information’s comparability was uncertain in two studies [19, 27], sensitivity analyses were conducted by removing these two studies for three primary outcomes, thought the results were not altered, which was neglected in previous meta-analyses [32–35].

In contrast to previous reviews [32–34], some large-scale studies included in our report were published in the last 3 years, and outcomes were evaluated with long-term follow-up (12–60 months). Hence, the results of our meta-analysis are of credibility and stability. The limitations of our meta-analysis were: (i) methodological quality of some studies included in the meta-analysis was poor (lack of blinding and description of randomization processes); (ii) the number of included studies and sample size was small; and (iii) definitions or evaluation criteria in different studies were not consistent.

## Conclusions

In conclusion, our meta-analysis suggests that LNF and LTF have equivalently good control of GERD and could result in a similar prevalence of patient satisfaction. It is not rational or advisable to abandon LNF based on current evidence when choosing a surgical procedure for GERD. More large-scale, multicenter, high-quality, RCTs with a longer duration of follow-up are required to further clarify the value of LNF and LTF.

## Abbreviations

BMI, body mass index; DSGV, division of short gastric vessels; EM, esophageal motility; GERD, gastro-esophageal reflux disease; HR, high risk; LES, lower esophageal sphincter; LNF, laparoscopic Nissen fundoplication; LR, low risk; LTF, laparoscopic Toupet fundoplication; NR, not report; RCTs, randomized controlled trials; RR, risk ratio; SMD, Standard mean difference; UR, Unclear risk

## References

[1] Vakil N, van Zanten SV, Kahrilas P, Dent J, Jones R (2006). The Montreal definition and classification of gastroesophageal reflux disease: a global evidence-based consensus. Am J Gastroenterol.

[2] El-Serag HB, Sweet S, Winchester CC, Dent J (2014). Update on the epidemiology of gastro-oesophageal reflux disease: a systematic review. Gut.

[3] Hsu YC, Yang TH, Hsu WL, Wu HT, Cheng YC, Chiang MF, Wang CS, Lin HJ (2010). Mosapride as an adjunct to lansoprazole for symptom relief of reflux oesophagitis. Br J Clin Pharmacol.

[4] Liang WT, Yan C, Wang ZG, Wu JM, Hu ZW, Zhan XL, Wang F, Ma SS, Chen MP (2015). Early and midterm outcome after laparoscopic fundoplication and a minimally invasive endoscopic procedure in patients with gastroesophageal reflux disease: a prospective observational study. J Laparoendosc Adv Surg Tech A.

[5] Spechler SJ (1992). Comparison of medical and surgical therapy for complicated gastroesophageal reflux disease in veterans. The Department of Veterans Affairs Gastroesophageal Reflux Disease Study Group. N Engl J Med.

[6] Geagea T (1991). Nissen fundoplication by laparoscopy. Union Med Can.

[7] Dallemagne B, Weerts JM, Jehaes C, Markiewicz S, Lombard R (1991). Laparoscopic Nissen fundoplication: preliminary report. Surg Laparosc Endosc.

[8] Ruiz-Tovar J, Diez-Tabernilla M, Chames A, Morales V, Martinez-Molina E (2010). Clinical outcome at 10 years after laparoscopic versus open Nissen fundoplication. J Laparoendosc Adv Surg Tech A.

[9] Hakanson BS, Thor KB, Thorell A, Ljungqvist O (2007). Open vs laparoscopic partial posterior fundoplication. A prospective randomized trial. Surg Endosc.

[10] Draaisma WA, Buskens E, Bais JE, Simmermacher RK, Rijnhart-de JH, Broeders IA, Gooszen HG (2006). Randomized clinical trial and follow-up study of cost-effectiveness of laparoscopic versus conventional Nissen fundoplication. Br J Surg.

[11] Korkolis DP, Kapritsou M, Konstantinou EA, Giannakopoulou M, Chrysi MS, Tsakiridou M, Kouloura A, Flamourakis M, Maricosu M, Gontikakis E, Plataniotis G (2015). The impact of laparoscopic Nissen fundoplication on the long-term quality of life in patients with gastroesophageal reflux disease. Gastroenterol Nurs.

[12] SAGES SOAG (1998). Guidelines for surgical treatment of gastroesophageal reflux disease (GERD). Surg Endosc.

[13] Owers C, Ackroyd R (2013). Management of gastroesophageal reflux disease and hiatus hernia: Overview and authors’ perspective. J Surg.

[14] Mickevicius A, Endzinas Z, Kiudelis M, Jonaitis L, Kupcinskas L, Maleckas A, Pundzius J (2008). Influence of wrap length on the effectiveness of Nissen and Toupet fundoplication: a prospective randomized study. Surg Endosc.

[15] Watson DI, Jamieson GG, Lally C, Archer S, Bessell JR, Booth M, Cade R, Cullingford G, Devitt PG, Fletcher DR, Hurley J, Kiroff G, Martin CJ, Martin IJ, Nathanson LK, Windsor JA (2004). Multicenter, prospective, double-blind, randomized trial of laparoscopic nissen vs anterior 90 degrees partial fundoplication. Arch Surg.

[16] Varin O, Velstra B, De Sutter S, Ceelen W (2009). Total vs partial fundoplication in the treatment of gastroesophageal reflux disease: a meta-analysis. Arch Surg.

[17] Ramos RF, Lustosa SA, Almeida CA, Silva CP, Matos D (2011). Surgical treatment of gastroesophageal reflux disease: total or partial fundoplication? systematic review and meta-analysis. Arq Gastroenterol.

[18] Ma S, Qian B, Shang L, Shi R, Zhang G (2012). A meta-analysis comparing laparoscopic partial versus Nissen fundoplication. ANZ J Surg.

[19] Qin M, Ding G, Yang H (2013). A clinical comparison of laparoscopic Nissen and Toupet fundoplication for gastroesophageal reflux disease. J Laparoendosc Adv Surg Tech A.

[20] Koch OO, Kaindlstorfer A, Antoniou SA, Luketina RR, Emmanuel K, Pointner R (2013). Comparison of results from a randomized trial 1 year after laparoscopic Nissen and Toupet fundoplications. Surg Endosc.

[21] Wang B, Zhang W, Liu S, Du Z, Shan C, Qiu M (2015). A Chinese randomized prospective trial of floppy Nissen and Toupet fundoplication for gastroesophageal disease. Int J Surg.

[22] Moher D, Liberati A, Tetzlaff J, Altman DG (2010). Preferred reporting items for systematic reviews and meta-analyses: the PRISMA statement. Int J Surg.

[23] DerSimonian R, Laird N (1986). Meta-analysis in clinical trials. Control Clin Trials.

[24] Higgins JP, Altman DG, Gotzsche PC, Juni P, Moher D, Oxman AD, Savovic J, Schulz KF, Weeks L, Sterne JA (2011). The Cochrane Collaboration’s tool for assessing risk of bias in randomised trials. BMJ.

[25] Chrysos E, Tsiaoussis J, Zoras OJ, Athanasakis E, Mantides A, Katsamouris A, Xynos E (2003). Laparoscopic surgery for gastroesophageal reflux disease patients with impaired esophageal peristalsis: total or partial fundoplication?. J Am Coll Surg.

[26] Guérin E, Betroune K, Closset J, Mehdi A, Lefebvre JC, Houben JJ, Gelin M, Vaneukem P, El NI (2007). Nissen versus Toupet fundoplication: results of a randomized and multicenter trial. Surg Endosc.

[27] Strate U, Emmermann A, Fibbe C, Layer P, Zornig C (2008). Laparoscopic fundoplication: Nissen versus Toupet two-year outcome of a prospective randomized study of 200 patients regarding preoperative esophageal motility. Surg Endosc.

[28] Booth MI, Stratford J, Jones L, Dehn TC (2008). Randomized clinical trial of laparoscopic total (Nissen) versus posterior partial (Toupet) fundoplication for gastro-oesophageal reflux disease based on preoperative oesophageal manometry. Br J Surg.

[29] Shaw JM, Bornman PC, Callanan MD, Beckingham IJ, Metz DC (2010). Long-term outcome of laparoscopic Nissen and laparoscopic Toupet fundoplication for gastroesophageal reflux disease: a prospective, randomized trial. Surg Endosc.

[30] Mughal MM, Bancewicz J, Marples M (1990). Oesophageal manometry and pH recording does not predict the bad results of Nissen fundoplication. Br J Surg.

[31] Liang WT, Wu JM, Hu ZW, Wang ZG, Zhu GC, Zhang C (2014). Laparoscopic Nissen fundoplication is more effective in treating patients with GERD-related chronic cough than Stretta radiofrequency. Minerva Chir.

[32] Shan CX, Zhang W, Zheng XM, Jiang DZ, Liu S, Qiu M (2010). Evidence-based appraisal in laparoscopic Nissen and Toupet fundoplications for gastroesophageal reflux disease. World J Gastroenterol.

[33] Broeders JA, Mauritz FA, Ahmed AU, Draaisma WA, Ruurda JP, Gooszen HG, Smout AJ, Broeders IA, Hazebroek EJ (2010). Systematic review and meta-analysis of laparoscopic Nissen (posterior total) versus Toupet (posterior partial) fundoplication for gastro-oesophageal reflux disease. Br J Surg.

[34] Tan G, Yang Z, Wang Z (2011). Meta-analysis of laparoscopic total (Nissen) versus posterior (Toupet) fundoplication for gastro-oesophageal reflux disease based on randomized clinical trials. ANZ J Surg.

[35] Tian ZC, Wang B, Shan CX, Zhang W, Jiang DZ, Qiu M (2015). A meta-analysis of randomized controlled trials to compare long-term outcomes of Nissen and toupet fundoplication for gastroesophageal reflux disease. PLoS One.

[36] Fein M, Bueter M, Thalheimer A, Pachmayr V, Heimbucher J, Freys SM, Fuchs KH (2008). Ten-year outcome of laparoscopic antireflux surgery. J Gastrointest Surg.

[37] Khajanchee YS, O’Rourke RW, Lockhart B, Patterson EJ, Hansen PD, Swanstrom LL (2002). Postoperative symptoms and failure after antireflux surgery. Arch Surg.

[38] Mardani J, Lundell L, Engstrom C (2011). Total or posterior partial fundoplication in the treatment of GERD: results of a randomized trial after 2 decades of follow-up. Ann Surg.

[39] Kamolz T, Granderath FA, Bammer T, Wykypiel HJ, Pointner R (2002). “Floppy” Nissen vs. Toupet laparoscopic fundoplication: quality of life assessment in a 5-year follow-up (part 2). Endoscopy.

[40] Catarci M, Gentileschi P, Papi C, Carrara A, Marrese R, Gaspari AL, Grassi GB (2004). Evidence-based appraisal of antireflux fundoplication. Ann Surg.

